# Antimonial Resistance in *Leishmania donovani* Is Associated with Increased *In Vivo* Parasite Burden

**DOI:** 10.1371/journal.pone.0023120

**Published:** 2011-08-01

**Authors:** Manu Vanaerschot, Simonne De Doncker, Suman Rijal, Louis Maes, Jean-Claude Dujardin, Saskia Decuypere

**Affiliations:** 1 Unit of Molecular Parasitology, Department of Parasitology, Institute of Tropical Medicine, Antwerp, Belgium; 2 Laboratory for Microbiology, Parasitology and Hygiene, Department of Biomedical Sciences, Antwerp University, Antwerp, Belgium; 3 Department of Internal Medicine, B.P. Koirala Institute of Health Sciences, Dharan, Nepal; Federal University of São Paulo, Brazil

## Abstract

*Leishmania donovani* is an intracellular protozoan parasite that causes visceral leishmaniasis (VL). Antimonials (SSG) have long been the first-line treatment against VL, but have now been replaced by miltefosine (MIL) in the Indian subcontinent due to the emergence of SSG-resistance. Our previous study hypothesised that SSG-resistant *L. donovani* might have increased *in vivo* survival skills which could affect the efficacy of other treatments such as MIL. The present study attempts to validate these hypotheses. Fourteen strains derived from Nepalese clinical isolates with documented SSG-susceptibility were infected in BALB/c mice to study their survival capacity in drug free conditions (non-treated mice) and in MIL-treated mice. SSG-resistant parasites caused a significant higher *in vivo* parasite load compared to SSG-sensitive parasites. However, this did not seem to affect the strains' response to MIL-treatment since parasites from both phenotypes responded equally well to *in vivo* MIL exposure. We conclude that there is a positive association between SSG-resistance and *in vivo* survival skills in our sample of *L. donovani* strains which could suggest a higher virulence of SSG-R strains compared to SSG-S strains. These greater *in vivo* survival skills of SSG-R parasites do not seem to directly affect their susceptibility to MIL. However, it cannot be excluded that repeated MIL exposure will elicit different adaptations in these SSG-R parasites with superior survival skills compared to the SSG-S parasites. Our results therefore highlight the need to closely monitor drug efficacy in the field, especially in the context of the Kala-azar elimination programme ongoing in the Indian subcontinent.

## Introduction

Visceral leishmaniasis (VL) is a major public health challenge in Sudan, Brazil, Bangladesh, India and Nepal where 90% of all VL cases occur, but is also prevalent in many other countries in Africa, South-America, Southern Europe and Asia [1]. In the Indian subcontinent, VL is caused by *Leishmania donovani* and is transmitted by sand flies. Since the disease is lethal if left untreated, treatment is one of the major pillars in the fight against VL. Pentavalent antimonials (SSG) are used worldwide as the first-line treatment against VL since many years, but ever increasing failure of SSG in some regions has urged the authorities of India, Nepal and Bangladesh to abandon this treatment and replace it with the oral drug miltefosine (MIL). The use of MIL in combination with early diagnosis and vector control are currently the major pillars of the Kala-azar elimination programme in the Indian subcontinent [2].

SSG-treatment failure in India and Nepal has been linked to SSG-resistant parasites [3], [4], but the exact mechanism of SSG-resistance is as yet unclear and presumably multifactorial [5]–[9]. SSG is known to reinforce the killing mechanisms of macrophages, the host cells of *Leishmania*, which contributes to the elimination of the intracellular parasites [8]. Hence, it is conceivable that SSG-resistant parasites have acquired superior skills to survive within the macrophage compared to SSG-sensitive parasites. Based on the results of our previous study in which SSG-sensitive and SSG-resistant model strains were characterised on different levels, we hypothesised that SSG-resistant strains are better equipped to survive *in vivo* compared to SSG-sensitive strains [10]. In the present study, this hypothesis was tested using a large sample of *L. donovani* strains which is representative for the parasite populations circulating in VL-endemic Nepal. In addition, the response to MIL-treatment of these strains with documented differential SSG-susceptibility was assessed in the BALB/c mouse model. The latter allowed evaluating whether the hypothesised superior *in vivo* survival skills of SSG-resistant parasites affect the efficacy of alternative treatments such as MIL.

## Materials and Methods

### 1. Ethical statements

Written informed consent was obtained from the patients and in the case of children, from the parents or guardians. The study was approved by the institutional ethical review boards of the Nepal Health Research Council, Kathmandu, Nepal, and the Institute of Tropical Medicine, Antwerp, Belgium.

Mouse care and experimental procedures were performed under approval of the Animal Ethic Committee of the Institute of Tropical Medicine Antwerp (approval ID PAR-018/2) and were in compliance with the national and international laws for the protection and welfare of animals.

### 2. Parasites

The *L. (L.) donovani* strains described in Table 1 were isolated from bone marrow aspirates from confirmed VL patients recruited at the B.P. Koirala Institute of Health Sciences, Dharan, Nepal [4]. The patients received a full supervised treatment course of sodium antimony gluconate (SAG) (Albert David Ltd, Calcutta) of 20 mg/kg/day i.m. for 30 days and were followed-up for clinical and parasitological evaluation at the end of treatment and at 3, 6 and 12 months after start of treatment. The clinical isolates were identified as *L. donovani* by PCR-RFLP analysis of cysteine proteinase b [11]. All isolates were cloned using the micro-drop method to obtain homogenous working parasite populations [12] and were subsequently tested for their SSG-susceptibility in an *in vitro* amastigote model exposing the parasite to 4 different SSG concentrations between 3 and 60 µg/ml SSG for 72 hours as described elsewhere [10]. The ED_50_ was then calculated using non-linear regression (sigmoidal dose response curve with variable slope). Every susceptibility test included the SSG-susceptible reference strain BPK206/0cl10 in order to determine the activity index of each strain (*i.e.* the ratio of the ED_50_ of the tested strain to the ED_50_ of the sensitive reference strain). A strain with an activity index of 3 or higher is considered to be SSG-resistant as described elsewhere [4].

**Table 1:** List of strains.

Name	International code	activity index to SSG	SSG-susceptibility
BPK282/0cl4	MHOM/NP/02/BPK282/0cl4	1	S
BPK294/0cl1	MHOM/NP/03/BPK294/0cl1	1	S
BPK043/0cl2	MHOM/NP/02/BPK043/0cl2	0	S
BPK091/0cl9	MHOM/NP/02/BPK091/0cl9	0	S
BPK206/0cl10	MHOM/NP/03/BPK206/0cl10	1	S
BPK080/0cl1	MHOM/NP/02/BPK080/0cl1	1	S
BPK298/0cl8	MHOM/NP/03/BPK298/0cl8	1	S
BPK178/0cl3	MHOM/NP/02/BPK178/0cl3	2	S
BPK190/0cl3	MHOM/NP/03/BPK190/0cl3	>6	R
BPK085/0cl3	MHOM/NP/02/BPK085/0cl3	>6	R
BPK275/0cl12	MHOM/NP/03/BPK275/0cl12	>6	R
BPK164/1cl11	MHOM/NP/02/BPK164/1cl11	>3	R
BPK173/0cl3	MHOM/NP/02/BPK173/0cl3	>6	R
BPK087/0cl11	MHOM/NP/02/BPK087/0cl11	3	R

All strains are derived from isolates taken before the onset of treatment, except for BPK164/1cl11 which is derived from an isolate taken at the end of treatment. The activity index to SSG is the ratio of the ED_50_ of a tested strain to the ED_50_ of the sensitive reference strain BPK206/0cl10. A strain with an activity index of 3 or higher is considered to be SSG-resistant as described elsewhere [4].

### 3. *In vivo* infections

*In vivo* infections were performed as described elsewhere [10]. Briefly, female BALB/c isogenic mice were infected with 10^7^ stationary phase promastigotes and randomly allocated into 42 groups of five (3 groups for each of the 14 strains). For the MIL-treatment study, mice were dosed orally at 10 mg/kg for 5 consecutive days, a dose known to cause 50% reduction in the hepatic parasite burden [13], starting at day 12 post-infection. MIL was kindly provided by Aeterna Zentaris GmbH (Germany). Mice were sacrificed at day 12 (non-treated group only) and day 20 (treated and non-treated group) post-infection to assess the parasite load in liver and spleen; two time points where the parasite burden showed to peak during *L. donovani* infection in previous experiments [10].

### 4. Quantification of parasite load and parasite response to *in vivo* MIL-treatment

Liver and spleen samples were collected and processed for quantification of *L. donovani* as described elsewhere [10]. In summary, DNA was extracted and assessed by 2 quantitative PCRs that were run on a LightCycler 480 (Roche): one targeting kDNA to quantify *Leishmania* and one targeting the single copy gene neurotrophin 3 of mice to quantify the amount of murine cells present in each sample. The parasite burden is described by a normalised amount of *Leishmania* per fixed unit of mouse cells, which allows comparison of parasite load between tissues. The resulting calibrated normalised relative quantities were calculated using qbase^plus^ (Biogazelle) [14] and exported to GraphPad Prism 5 (GraphPad Software) to determine the area under the infection curve (day 12 to day 20), a measure for the total parasite burden between day 12 and day 20 in infected BALB/c mice. The efficacy of MIL-treatment was evaluated by calculating the percentage of parasite clearance, *i.e.* the liver parasite load at day 20 of the MIL-treated group versus the non-treated control group.

## Results

### 1. *In vitro* SSG-susceptibility

Firstly, the fourteen *L. donovani* strains derived from Nepalese clinical isolates were tested for their SSG-susceptibility: 8 strains were typed as *in vitro* sensitive to SSG (SSG-S) and 6 strains showed to be *in vitro* resistant to SSG (SSG-R) (Table 1).

### 2. *In vivo* parasite burden

**Figure 1:**
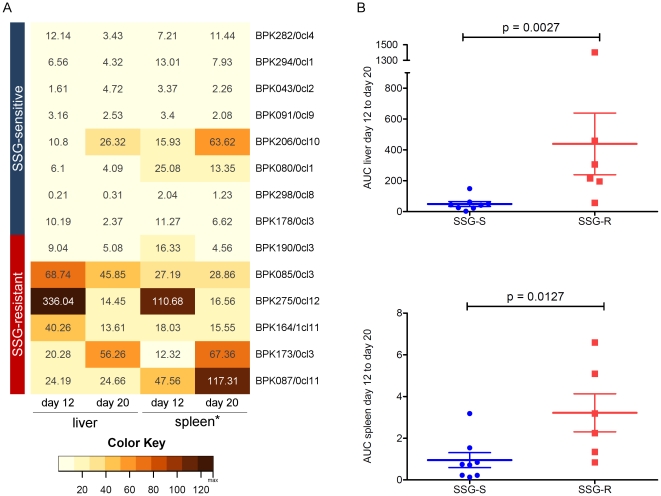
*In vivo* parasite load in liver and spleen of BALB/c mice caused by SSG-R and SSG-S strains. (A) Heat map showing mean parasite load in the liver and spleen* of infected mice at day 12 and day 20 post infection. *: spleen values were 100-times multiplied to allow easy comparison with liver in the heat map. (B) Total *in vivo* parasite load in infected mice calculated from the area under the day 12 to day 20 infection curve (AUC). P-values were calculated using the Mann Whitney U test. Horizontal bars indicate the mean and error bars indicate the standard error of the mean. The SSG-S strains show a homogenous infection profile in both the liver and spleen of infected BALB/c mice. Even though some variation is observed in the SSG-R group, analyses on both datasets indicate that SSG-R parasites cause a significant higher parasite burden in BALB/c mice compared to SSG-S parasites.

In a previous study, SSG-R model strains showed to cause higher *in vivo* parasite burdens in BALB/c mice compared to the SSG-S-strain [10]. In the current validation study, all SSG-S strains caused a similar parasite burden both in the liver and the spleen (Fig. 1). In contrast, SSG-R strains displayed a much more heterogeneous infection profile and an average 8-fold higher parasite burden in the liver and 3-fold higher parasite burden in the spleen compared to SSG-S strains (Fig. 1). Important to note is that the infection level in the spleen followed similar trends as in the liver, but was in average around 100 fold lower. All data is available in supporting information file Data S1.

### 3. *In vivo* efficacy of MIL-treatment

**Figure 2:**
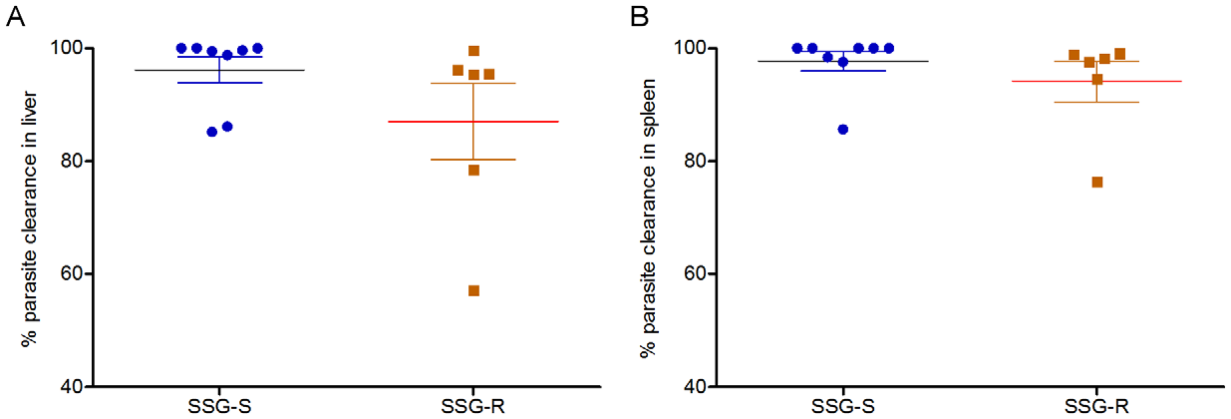
Percentage of miltefosine induced parasite clearance in the liver (A) and spleen (B) of infected BALB/c mice. In general, mice infected with SSG-S and SSG-R strains do not differ significantly in their response to miltefosine treatment (p>0.05, Mann Whitney U test). Horizontal bars indicate the mean and error bars indicate the standard error of the mean.

To assess whether or not the higher infection burden caused by SSG-R strains also affects their response to *in vivo* MIL-treatment, infected mice were treated orally with MIL. In general, SSG-R and SSG-S strains responded similarly to *in vivo* MIL-treatment (mean ± SEM: 96.14±2.30% clearance for the SSG-S group compared to 87.02±6.71% clearance for the SSG-R group in the liver and 97.70±1.75% clearance for SSG-S strains versus 94.09±3.62% clearance for SSG-R strains in the spleen), although some outliers in both groups were observed (Fig. 2). All data is available in supporting information file Data S1.

## Discussion

In the present work, we tested the hypothesis that SSG-R *L. donovani* have a better chance for survival *in vivo* compared to SSG-S *L. donovani* [10]. Results obtained with the current sample of 14 clinical *L. donovani* strains strengthen the hypothesis.

SSG-R strains showed a more variable and markedly higher capacity to cause *in vivo* infection compared to SSG-S strains, hereby suggesting a higher virulence of SSG-R strains. Even taking the limitations of a VL mouse model into account, these findings are in agreement with a study that showed an increased parasite burden in VL patients that eventually showed no response to SSG-treatment [15]. In another study, we also characterised the metacyclogenic capacity (a differentiation process essential for transmission and infectivity) of most of the strains which were analysed here. We could demonstrate that SSG-R strains develop on average twice as many metacyclic infectious parasites compared to SSG-S strains [16]. The higher metacyclogenic capacity of SSG-R strains could explain at least partly the characteristic higher parasite burden of SSG-R strains observed here. However, additional traits at the intracellular amastigote level might very well be co-responsible to maintain this initial advantage at the start of infection. Host cell manipulation is a well-known trait of *L. donovani* and a recent study demonstrated that SSG-R *L. donovani* specifically evolved extra mechanisms to manipulate its host cell to avoid SSG-induced stress [17]. Such adaptations would not only allow the parasites to survive better under SSG-pressure, but would also favour their survival in drug free conditions as observed here *in vivo.* The higher virulence of natural SSG-R *L. donovani* strains could very well contribute their general fitness. This is in contrast to *in vitro* induced pentamidine- or glibenclamide-resistant *L. mexicana* strains which had respectively a similar or decreased infectivity compared to the wild type [18], [19]. We cannot exclude that *L. mexicana* behaves differently compared to *L. donovani* under SSG-drug pressure. However, we believe that the differences between our study and to the 2 models resistant to other drugs discussed above might be due to the ‘host cell manipulation’ mode of action specific for SSG. In addition, we report on natural resistance which could differ significantly from *in vitro* induced resistance [20].

In a second part of the study, we tested whether the higher *in vivo* survival capacity of SSG-R strains could threaten the long term efficacy of other drugs. In theory, the higher the parasite load in one particular patient, the harder it is to clear the parasite load in that patient by drug treatment [15]. We comparatively evaluated the efficacy of MIL to clear SSG-S and SSG-R strain infections in BALB/c mice: SSG-R and SSG-S strains seemed to be equally well cleared by MIL from the spleen and liver of infected mice. This is in agreement with the *in vitro* MIL-susceptible phenotype of the parent isolates of which some of these strains were derived [21]. These findings are also consistent with clinical reports of successful MIL-treatment of SSG-failure patients during a phase-2 trial of MIL in India [22]. Indian studies did show that some natural SSG-S strains tend to have a higher *in vitro* tolerance to MIL compared to SSG-S strains [23] and some strains were successfully made resistant to MIL by *in vitro* exposure in the lab [13], [24]–[26]. However, importantly, natural MIL-R strains have not been identified in the field yet (unpublished results).

To conclude, our results suggest that SSG-R *L. donovani* have a higher virulence compared to SSG-S *L. donovani*. Whether or not this can have an impact on the long term efficacy of MIL in the Indian subcontinent is not entirely clear from our data on single exposure to MIL. Therefore, monitoring the efficacy of MIL in regions were SSG-R strains were reported to be highly prevalent is of the upmost importance for the current Kala-azar elimination programme to undertake swift action and switch to new treatment regimens [27] if necessary.

## Supporting Information

Data S1 Strain specific data of *in vivo* infections in untreated and MIL-treated mice.
(XLS)
